# G Protein-Coupled Receptor Heteromers as Putative Pharmacotherapeutic Targets in Autism

**DOI:** 10.3389/fncel.2020.588662

**Published:** 2020-10-30

**Authors:** Jon DelaCuesta-Barrutia, Olga Peñagarikano, Amaia M. Erdozain

**Affiliations:** ^1^Department of Pharmacology, University of the Basque Country (UPV/EHU), Leioa, Spain; ^2^Centro de Investigación Biomédica en Red en Salud Mental (CIBERSAM), Leioa, Spain

**Keywords:** GPCR receptor heteromers, pharmacotherapy, glutamate, oxytocin, serotonin, dopamine, ASD, cannabinoid

## Abstract

A major challenge in the development of pharmacotherapies for autism is the failure to identify pathophysiological mechanisms that could be targetable. The majority of developing strategies mainly aim at restoring the brain excitatory/inhibitory imbalance described in autism, by targeting glutamate or GABA receptors. Other neurotransmitter systems are critical for the fine-tuning of the brain excitation/inhibition balance. Among these, the dopaminergic, oxytocinergic, serotonergic, and cannabinoid systems have also been implicated in autism and thus represent putative therapeutic targets. One of the latest breakthroughs in pharmacology has been the discovery of G protein-coupled receptor (GPCR) oligomerization. GPCR heteromers are macromolecular complexes composed of at least two different receptors, with biochemical properties that differ from those of their individual components, leading to the activation of different cellular signaling pathways. Interestingly, heteromers of the above-mentioned neurotransmitter receptors have been described (e.g., mGlu2–5HT2A, mGlu5–D2–A2A, D2–OXT, CB1–D2, D2–5HT2A, D1–D2, D2–D3, and OXT–5HT2A). We hypothesize that differences in the GPCR interactome may underlie the etiology/pathophysiology of autism and could drive different treatment responses, as has already been suggested for other brain disorders such as schizophrenia. Targeting GPCR complexes instead of monomers represents a new order of biased agonism/antagonism that may potentially enhance the efficacy of future pharmacotherapies. Here, we present an overview of the crosstalk of the different GPCRs involved in autism and discuss current advances in pharmacological approaches targeting them.

## Introduction

Autism spectrum disorder (ASD) is a severe developmental disorder that involves difficulties in two behavioral domains: social interaction, including speech and nonverbal communication, and restricted/repetitive behaviors [American Psychiatric Association (Ed.), 2013]. These core symptoms are frequently associated with other emotional and behavioral disturbances, such as anxiety, irritability, inattention, hyperactivity, and sleep problems, resulting in a very heterogeneous clinical manifestation. The etiology of ASD is also complex, caused by a combination of genetic (~80%) and environmental factors (Bai et al., 2019). The causal neuropathology is largely unknown. As a result, there are currently no medications approved for the management of the core symptoms of ASD; however, most affected individuals follow pharmacological interventions to target associated symptoms, albeit with limited evidence-based efficiency and substantial adverse effects.

Research aimed at developing targeted pharmacotherapies for ASD identifies functional alterations in brain areas and networks involved in emotion and social cognition, such as the prefrontal cortex (PFC) and limbic system (Kennedy and Adolphs, 2012; Ecker et al., 2015; Fernández et al., 2018; Müller and Fishman, 2018). At the molecular level, an alteration in several neurotransmitter systems that modulate the activity of these brain areas and networks has been observed. The imbalance between excitatory glutamatergic and inhibitory GABAergic tones has been the most studied (Uzunova et al., 2016). Hence, one strategy that aimed at restoring this imbalance relies on antagonizing glutamate receptors (e.g., memantine) or using GABA agonists (e.g., arbaclofen), in order to reduce the proposed overstimulated glutamate signaling in ASD (Rojas, 2014; Fernández et al., 2018). However, hypo-glutamate theories have also been proposed, and glutamate receptor agonists such as D-cycloserine are also used in some cases, indicating that the excitatory/inhibitory imbalance might occur in both directions (Fernández et al., 2018). Nonetheless, the oxytocinergic (OXT), serotonergic (5HT), dopaminergic (DA), and cannabinoid (CB) systems are also critical for the fine-tuning of the brain excitation/inhibition balance and thus represent putative therapeutic targets in ASD (Marotta et al., 2020).

Once the role of the OXT system in modulating affiliative and social behavior across vertebrate species was established, efforts in translating these findings to the clinic begun (Insel, 2010; Yamasue and Domes, 2017; Erdozain and Peñagarikano, 2020). Alterations in OXT receptor (OXTR) binding and OXTR gene hypermethylation have been found in different brain structures of individuals with ASD (Purba, 1996; Lee et al., 2007). Several studies have also reported altered plasma OXT levels (Modahl et al., 1998; Andari et al., 2010; Aydın et al., 2018; Strauss et al., 2019), albeit there is still much debate on whether peripheral OXT correlates with the one in brain (Jupiter et al., 1988). An increasing number of clinical trials testing the effect of OXT or OXT agonists in ASD are being carried out. Although based on current results OXT seems to have a potential therapeutic value, there are key questions that remain unanswered as to decide the optimal target groups and treatment course (Erdozain and Peñagarikano, 2020).

Regarding 5HT, hyperserotonemia was the first blood biomarker proposed in ASD, as it is present in more than 25% of affected children (Hanley, 1977; Cook, 1990). Since then, many studies have observed changes in the 5HT system in ASD. One example is the decrease of 5HT receptor 2A (5HT2AR) binding detected by imaging studies (Murphy et al., 2006). In postmortem studies, lower binding of 5HT receptor 1A (5HT1AR), in addition to 5HT2AR, has also been reported (Oblak et al., 2013), suggesting a deficient 5HT signaling in brain, albeit the presence of blood hyperserotonemia. Accordingly, selective serotonin reuptake inhibitors (SSRIs) have long been used to treat symptoms of repetitive behavior and anxiety in autism, although with limited clinical efficacy (King et al., 2009).

There are promising studies about the influence of the CB system in ASD due to CB’s pro-social properties and its interaction with the OXT system. Lower serum endocannabinoid levels have been reported in children with ASD (Aran et al., 2019), and there is initial evidence of its effectiveness in improving ASD comorbidities such as self-injury, hyperactivity, and anxiety (Poleg et al., 2019).

Last, dysfunction in the DA system has also been observed in ASD, although with apparently conflicting results, with some studies reporting an overstimulation and others a downregulation of DA transmission. To conceal these results, a DA hypothesis of ASD has been proposed, in which upregulation of the DA nigrostriatal pathway would lead to the stereotypic/repetitive behaviors in ASD, while downregulation of the mesocorticolimbic pathway would lead to social deficits (Pavăl, 2017). The relevance of the DA system in ASD is evident by the fact that the only Food and Drug Administration (FDA)-approved pharmacotherapy to treat associated symptoms in ASD, such as irritability and aggression, are atypical antipsychotics (i.e., risperidone and aripiprazole) that antagonize D2R, albeit they show significant metabolic adverse effects (De Hert et al., 2011).

**Figure 1:**
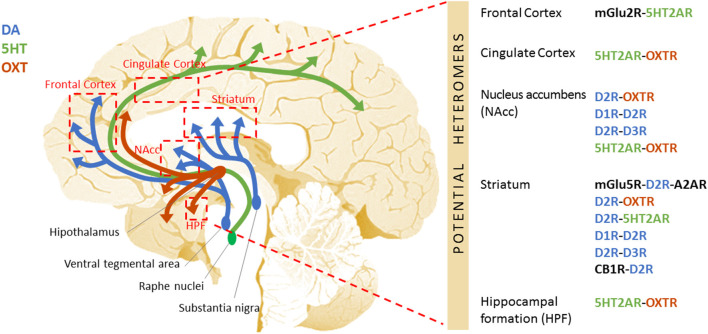
Schematic representation of the potential GPCR heteromers proposed as new therapeutic targets to treat autism. Aside from the widely studied GABAergic and glutamatergic systems, other neurotransmitter systems are critical for the fine-tuning of the excitatory/inhibitory imbalance described in autism. Among these are the dopaminergic, serotonergic, oxytocinergic, and cannabinoid systems. The specific receptors for these neurotransmitters equally represent putative therapeutic targets. Further, these GPCRs heteromers have been shown to display properties that differ from those of their individual components, leading to the activation of distinctive cellular signaling pathways. Differences in the GPCR interactome may underlie the etiology/pathophysiology of autism. This figure shows the DA (blue), 5HT (green), and OXT (red) neural circuits, and the brain regions they connect to (red boxes) where the existence of GPCR heteromers has been described in mammalian brain (mouse, rat, primate, or human). On the right side, the specific GPCR heteromers found in each of these regions are specified.

The scarceness of effective therapeutic treatments for autism and its comorbidities are startling, and the undesirable effects of the currently prescribed drugs abundant. Therefore, an important effort is being made to identify new putative therapeutic targets (Famitafreshi and Karimian, 2018). Oligomerization of G protein-coupled receptors (GPCRs) is one of the latest breakthroughs in pharmacology and could be one of the keys to overcome this therapeutic barrier. In this review, we first introduce the concept of GPCR heteromers. Second, we present different GPCR heteromers that could be of interest as putative pharmacotherapeutic targets in autism. Last, we present some pharmacological tools that are already available to modulate them (Figure 1).

## G Protein-Coupled Receptor Heteromers as Pharmacological Targets

### Heteromer History

GPCR are seven-transmembrane (TM) domain proteins involved in cell-to-cell signalization and are the target of 30–40% of current pharmaceutical drugs (Albizu et al., 2010). While oligomerization is a common biological process, the concept of GPCR oligomerization was not introduced until the 1980’s (Agnati et al., 1980, 1982; Birdsall, 1982; Avissar et al., 1983). A relevant historic episode was the discovery of GABA_B_ receptor dimerization, an obligate receptor dimer (Jones et al., 1998; Kaupmann et al., 1998; Kuner et al., 1999; White et al., 1998; Margeta-Mitrovic et al., 2000). Since then, several receptor homomers and heteromers have been discovered in the central nervous system (Moreno et al., 2013). Oligomerization exerts significant impact on receptor function and physiology, offering a platform for the diversification of receptor signaling, pharmacology, regulation, crosstalk, internalization, and trafficking (Farran, 2017). Therefore, heteromers could constitute important therapeutic targets for a wide range of disorders, including ASD.

### Heteromer Definition Criteria

Receptor heteromers are oligomeric complexes composed of at least two functional receptor units (i.e., protomers), which interact with each other through the TM domains and show different biochemical properties from those of their individual components (Ferré et al., 2009; Gomes et al., 2016). The International Union of Basic and Clinical Pharmacology proposed five recommendations for the recognition and acceptance of receptor heteromers in the scientific community, and at least two of these criteria should be met for this designation: (1) evidence for physical association in native or primary cells; (2) colocalization of the protomers within the same subcellular compartment in the same cell; (3) proof of the physical interaction between the two receptor protomers in native tissue using coimmunoprecipitation experiments, energy transfer technologies, or transgenic animals expressing physiological levels of recombinant fluorescent proteins; (4) identification of a unique pharmacological property specific of the heteromer; and (5) demonstration of *in vivo* heteromerization using knockout animals or RNAi technology.

## Putative G Protein-Coupled Receptor Heteromers Implicated in Autism

**Figure 2:**
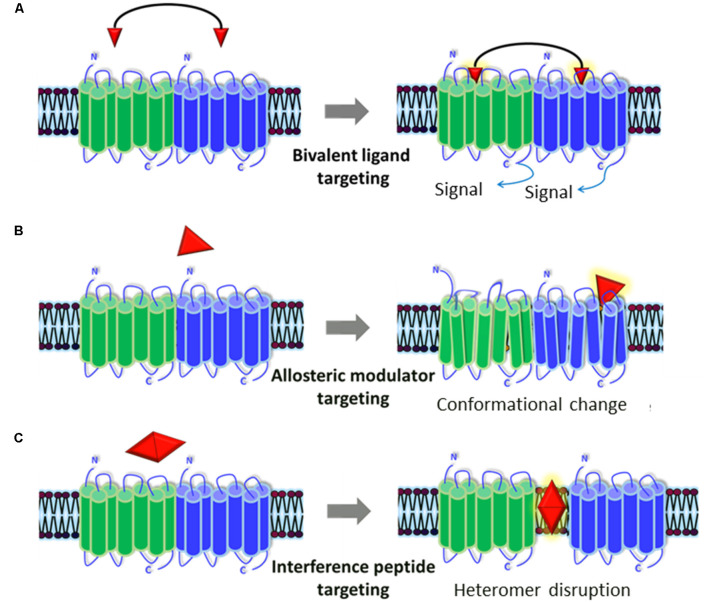
Representation of the pharmacological tools to modulate GPCR heteromers. Currently available pharmacological tools to modulate the generation and/or signaling of receptor heteromers could represent new therapeutic strategies for ASD. **(A)** Bivalent ligands are composed of two functional pharmacophores, linked by a spacer, that interact with each of the protomers of the heteromers and activate/block its cellular signaling cascade(s). **(B)** Allosteric modulators bind to distinct binding sites from the endogenous ligands in one of the protomers and can alter its structure (producing a conformational change), dynamics, and function, which will in turn alter the whole heteromer’s functionality. **(C)** Interference peptides are synthetic peptides harboring the same amino acid sequence as the interacting transmembrane (TM) domains between the two receptors that compose the heteromer: they get inserted into TM domains and disrupt the receptor heteromer by preventing binding between the two receptor protomers.

Several GPCR heteromers containing receptors involved in the etiology/pathophysiology of autism, including glutamatergic, DA, OXT, and 5HT receptors, have been described. Dysfunction in the formation and/or function of such heteromers could potentially contribute to the disorder, as has already been suggested for other brain disorders such as schizophrenia. These GPCR heteromers might, thus, represent new pharmacotherapeutic targets for autism (Figure 2).

### mGlu2R–5HT2AR

mGlu2R and 5HT2AR have been shown to colocalize and interact with each other in mouse and human frontal cortex (Delille et al., 2013; Moreno et al., 2016). Although mGlu2R is coupled to Gi/o proteins and 5HT2AR to Gq/11, it has been shown that acting through the mGlu2R–5HT2AR heterocomplex, both 5HT and glutamatergic ligands modulate Gq/11- and Gi/o-dependent signaling (Fribourg et al., 2011). The pathophysiological role of the heterocomplex has been mainly studied in relation to psychosis and schizophrenia, where altered signaling through this heteromer has been observed (González-Maeso et al., 2008; Moreno et al., 2016; Shah and González-Maeso, 2019). Further, observations in mice and cultured cells suggest that the mGluR2–5HT2AR complex, and not 5HT2AR alone, is the molecular target responsible for the actions of hallucinogenic drugs such as lysergic acid diethylamide (LSD) and that activation of mGlu2R abolishes hallucinogen-specific signaling and behavioral responses (González-Maeso et al., 2008; Moreno et al., 2011; Halberstadt et al., 2019). Although so far no study has assessed the role of mGlu2R–5HT2AR heteromer in ASD, its association with schizophrenia, a disorder that shares some risk factors and displays overlapping traits with ASD, including neuroimaging evidence and mutual comorbidity (Chisholm et al., 2015), indicates that its evaluation would be worthwhile. Thus, the evaluation of mGlu2R–5HT2AR complex in ASD animal models and/or postmortem human brain could bring new approaches to understand the disorder and find new pharmacological targets.

### mGlu5R–D2R–A2AR

The existence of mGlu5R–D2R–A2AR oligomers has been reported in native rat striatum and GABAergic striatopallidal neurons, where they are mainly formed (Simola et al., 2008; Cabello et al., 2009; Lewis et al., 2019). This heteromer might be of relevance in ASD due to its putative role in repetitive behavior and reward. A recent study has evaluated the potential effect of different drug combinations on the repetitive behaviors characteristic of deer mice: no single drug or double-drug combinations were effective, albeit the combination of a D2R antagonist, an A2AR agonist, and an mGlu5R positive allosteric modulator reduced repetitive behaviors. In contrast, the combination of a D2R agonist, an A2AR antagonist, and an mGlu5R negative allosteric modulator caused a significant increase in repetitive behavior (Lewis et al., 2019). Regarding the reward system, the antidepressant basimglurant, an mGlu5R negative allosteric modulator, has been proposed to reduce the anti-reward effect exerted by GABA neurons of the ventral striatopallidal pathway, because it inhibits D2R signaling in A2A–D2–mGlu5 receptor heteromers (Fuxe and Borroto-Escuela, 2015). This D2R signal reduction in striatopallidal GABA neurons has also been reported with a combination of an mGlu5R antagonist and an A2AR antagonist (Beggiato et al., 2016). Thus, selectively modulating the functionality of this oligomer through any of its protomers could constitute a novel approach to treat repetitive behaviors in ASD and to improve social interaction through the reward system.

### D2R–OXTR

D2R–OXTR heteromers’ existence has been revealed in the nucleus accumbens (NAcc) and dorsal striatum of female prairie voles, a species widely used to investigate social behavior (Fuxe et al., 2014). In terms of physiology, radio-ligand experiments in voles with NAcc membrane preparations demonstrated that OXT very significantly modified the affinity of D2R antagonists and agonists, an effect that was blocked by an OXTR antagonist (Fuxe et al., 2014). The co-activation of D2R and OXTR in NAcc has been reported to be necessary for pair bond formation in female voles (Baskerville and Douglas, 2010). Moreover, OXT administration increases NAcc DA release and improves social behavior in rats (Kohli et al., 2019). Hence, difficulties in developing social bonds in ASD could be possibly improved by the activation of D2R–OXTR heteromer in NAcc. In addition, OXT infusion into the central amygdala elicited anxiolytic effects in rats, which was prevented with a simultaneous infusion of a D2/D3 antagonist, suggesting the involvement of the D2R–OXTR heteromer in this brain region. In consequence, OXT’s potential benefit for reducing anxiety in ASD may rely on targeting the D2R–OXTR heteromer, possibly in the amygdala.

### CB1R–D2R

The presence of the CB1R–D2R heteromer has been recently reported in the globus pallidus in mice (Bagher et al., 2020). The heteromer activation is characterized by an antagonistic interaction between the protomers, with CB1R agonists reducing the affinity and hyper-locomotor activity exerted by D2R agonists (Marcellino et al., 2008). For this reason, targeting CB1R–D2R with CB1R agonists in striatum may be a pharmacological alternative to treat irritability or hyperactivity in ASD. In addition, immunoelectron microscopy suggests that, in mice, CB1R and D2Rs colocalize in GABAergic terminals of the PFC, in which activation of either receptor could suppress GABA release onto layer 5 pyramidal cells (Chiu et al., 2010). Thus, targeting CB1R–D2R might also represent a novel strategy to improve the excitatory/inhibitory imbalance in ASD.

### D2R–5HT2AR

Existence of 5HT2AR–D2R heteromers has been demonstrated in rat striatum (Borroto-Escuela et al., 2014). The heteromer displays bidirectional receptor–receptor interaction, D2R agonists increasing hallucinogenic agonists’ affinity for 5HT2AR (Albizu et al., 2011), and *vice versa*, hallucinogenic 5HT2AR agonists increasing D2R density (Borroto-Escuela et al., 2014). Thus, the simultaneous antagonization of both protomers has been proposed as an antipsychotic strategy, with fewer side effects and lower doses required (Borroto-Escuela et al., 2014; Zhang et al., 2020). In fact, the advantageous extrapyramidal side-effect profile of the atypical antipsychotic risperidone, which behaves as a D2R and 5HT2A antagonist and is one of the most widely used drugs to treat associated symptoms in ASD, would rely on targeting the dimer and/or inducing its oligomerization (Borroto-Escuela et al., 2014; Kolasa et al., 2018). In agreement with this, a recent computational model of 3D structure–activity relationship of D2R and 5HT2AR antagonists reported that targeting the heteromer simultaneously significantly reduced extrapyramidal side effects of antipsychotic treatment (Zhang et al., 2020).

### D1R–D2R

D1R–D2R heteromer was demonstrated in rat and nonhuman primate NAcc (Perreault et al., 2016; Rico et al., 2017) and in human striatum (Pei et al., 2010). The pathophysiological role of this heteromer has been largely studied in depression and anxiety, which are two of the most common comorbidities in ASD (Simonoff et al., 2008). D1R–D2R heteromer formation was found to be increased in human postmortem striatum of subjects with major depression (Pei et al., 2010), and disrupting the dimer in rats produced antidepressant (Hasbi et al., 2014) and anxiolytic effects (Shen et al., 2015). A recent study reported overexpressed D1R–D2R heteromers in female nonhuman primate and rat brain, along with higher depressive-like and anxiety-like behaviors, which are improved by disruption of the dimer (Hasbi et al., 2020). In consequence, D1R–D2R heteromer disruption might be an alternative treatment for individuals with depressive or anxiety symptoms in ASD and potentially more effective in female subjects. Further, the D1R–D2R heteromer might also modulate social behavior through the reward pathway, as disrupting this heteromer in the NAcc has been shown to increase the rewarding effects of drugs of abuse in rats (Perreault et al., 2016).

### D2R–D3R

Colocalization of D2R and D3R was detected in the globus pallidus and NAcc of rats (Surmeier et al., 1997), and their interaction was determined by co-immunoprecipitation studies in cultured cells (Scarselli et al., 2001). A putative role for the D2R–D3R heteromer as a target for antipsychotics has been suggested, since antipsychotics with partial D2R agonist properties act as D2R antagonists in the presence of the dimer (Maggio and Millan, 2010). This has strong implications for ASD as aripiprazole, one of the most widely used antipsychotics to treat irritability in autism (Goel et al., 2018), is a partial D2R agonist that would act as antagonist in brain areas where the heterodimer is expressed, gaining brain region-specific effects (Maggio and Millan, 2010). This property of aripiprazole has been proposed to account for the fewer extrapyramidal effects elicited by this drug (Maggio et al., 2015).

### 5HT2AR–OXTR

Despite the abundant evidence across species of the interaction between the OXT and 5HT systems in the regulation of socio-cognitive behaviors (Lefevre et al., 2017; Nagano et al., 2018; Tan et al., 2020), anxiety (Yoshida et al., 2009), and reward (Aubert et al., 2013; Dölen et al., 2013), only one recent report has provided evidence of the existence of a 5HT2AR–OXTR heterodimer, which was detected in in rat hippocampus, cingulate cortex, and NAcc, key regions associated with cognition and the above-described behaviors (Chruścicka et al., 2019). The authors, using functional cellular-based assays, proved that the 5HT2AR–OXTR heterocomplex formation leads to bidirectional antagonistic receptor−receptor interactions, reducing Gαq signaling (Chruścicka et al., 2019). The implication of this recently identified heteromer in a pathological state has not been evaluated yet, but it might potentially be an interesting target to improve social behavior and anxiety in ASD.

## Pharmacological Tools to Modulate Receptor Heteromers

Currently, there are several pharmacological tools that allow the modulation of receptor heteromers. These include bivalent ligands, allosteric modulators, and interference peptides.

### Bivalent Ligands

Bivalent ligands are composed of two functional pharmacophores, linked by a spacer, each with potential of interacting with a protomer of the dimeric receptor (Berque-Bestel et al., 2008). Bivalent ligands of the μ opioid receptor (μOR) were the first to be developed, demonstrating that stimulation of the heteroreceptors δOR-μOR, μOR-CB1R, or μOR-mGlu5R reduced nociception to a bigger extent than morphine and without some of its adverse effects such as tolerance, dependence, and respiratory depression (Gomes et al., 2016; Machelska and Celik, 2018). Hence, using bivalent ligands to target the heteromers potentially affected in ASD, as discussed above, could be more efficient than targeting the receptors separately. For instance, antagonist bivalent ligands targeting D2R–5HT2AR or D2R–D3R heteromers could be an alternative to current antipsychotics to improve irritability, and agonist bivalent ligands targeting D2R–OXTR could be beneficial for social behavior.

### Allosteric Modulators

Allosteric sites of GPCR are distinct from binding sites for endogenous ligands and can alter the receptor structure, dynamics, and function in order to achieve a potential therapeutic advantage (Hauser et al., 2017). Positive allosteric modulators increase the effect of agonists, in contrast to negative allosteric modulators that inhibit their effect. Selective modulators of receptor heteromers could expand the range of therapeutic options to treat autism. For instance, mGlu5R positive allosteric modulator targeting mGlu5R–D2R–A2AR heteromer in combination with other drugs, such as a D2R antagonist and an A2AR agonist, as described above (Lewis et al., 2019), may improve repetitive behavior symptoms in ASD.

### Interference Peptides

Interference peptides are synthetic peptides harboring the same amino acid sequence as the interacting TM domains between the two receptors that compose the heteromer (Botta et al., 2019). These peptides harbor a small signal peptide that facilitates penetration in the cell. They get inserted into TM domains, disrupting the receptor heteromer by preventing binding between the two receptor protomers. Interference peptides have contributed to validate and understand the functional consequences of receptor oligomerization and could become an alternative to treat ASD. As above mentioned, disrupting D1R–D2R receptor heteromer could be beneficial for individuals with autism due to its anxiolytic and antidepressant effect (Hasbi et al., 2014; Shen et al., 2015). Nevertheless, more efficient interference peptides need to be developed, since their *in vivo* efficacy is often compromised by the loss of secondary structure, deficient cellular penetration, and susceptibility to proteolysis in the digestive system (Botta et al., 2019).

## Conclusion and Future Directions

There is no doubt that alterations in the GABAergic, glutamatergic, OXT, 5HT, DA, and CB systems are associated with ASD. In an attempt to search for novel targeted therapeutic approaches to treat ASD and overcome the scarceness of effective pharmacological treatments, we propose a potential role for heterocomplexes formed by different receptors of these neurotransmitters. Oligomerization of GPCRs is one of the latest breakthroughs in pharmacology and could be one of the keys to overcome this therapeutic barrier. There is increasing evidence for the role of GPCR heteromers in the pathophysiology of other neuropsychiatric disorders, such as depression or schizophrenia, albeit no study has yet addressed this issue in autism. Postmortem brain samples provide a unique opportunity to advance molecular research in this regard (McCullumsmith et al., 2014). For example, coupling between dopamine D1 and D2 receptors was markedly increased in postmortem brain of subjects suffering from major depression, as detected by co-immunoprecipitation experiments (Pei et al., 2010). Blue native polyacrylamide gel electrophoresis is also a useful technique for this aim, as has already been used to observe alterations in receptor complexes in human brain for other diseases (Falsafi et al., 2016). Further, functional scintillation proximity assays for [35S]GTPγS binding have revealed dysregulated signaling *via* the mGlu2R–5HT2AR heteromer in postmortem human brain samples of schizophrenia subjects (Moreno et al., 2016). Once a potential molecular alteration is identified, existing pharmacological strategies to selectively modulate these GPCR heteromers could be tested to ascertain their potential to become new therapeutic treatments to improve some symptoms of ASD. Animal models provide a useful tool for mechanistic and behavioral outcome measures to address this issue. There are several well-validated animal models of ASD available (Möhrle et al., 2020), in which the expression of the above-mentioned GPCR heteromers could be quantified. In case that any of the complexes are found to be altered compared with those in control mice, a modulation of the heteromer function could be attempted using the modifiers described above, in order to assess whether it drives a behavioral improvement. In conclusion, the integrative use of ASD animal models and clinical subjects to understand the potential role of GPCR heteromers as mechanistic cause and putative pharmacological targets could open a new direction in understanding and treating ASD.

## Author Contributions

JD-B drafted the article. AE and OP contributed equally in the conception, design and supervision of the work. All authors contributed adding critical information in the different sections and helped shape the manuscript and design the figures. All authors contributed to the article and approved the submitted version.

## Conflict of Interest

The authors declare that the research was conducted in the absence of any commercial or financial relationships that could be construed as a potential conflict of interest.
